# Supplementary material for: Telomerase biogenesis requires a novel Mex67 function and a cytoplasmic association with the Sm7 complex
Source: eLife. 2020 Oct 23;9:e60000. doi: 10.7554/eLife.60000 (PMC7644208; doi:10.7554/eLife.60000)
Supplement: Supplementary file 1. — The table includes the names, genotypes and sources of all yeast strains used in the study. The yeast strains are referred to in the text using the names provided in the table. [file elife-60000-supp1.docx]

## Supplementary File 1. Yeast strains used in the study

| **Strain** | **Genotype** | **Source** |
| --- | --- | --- |
| W3749-1A | *Mat A, ura3-1, his3-11,15, leu2-3,112, trp1-1, ade2-1, bar1::LEU2* | (Lisby *et al.*, 2004) |
| YV347, YV348 | *Mat A, ura3-1, his3-11,15, leu2-3,112, trp1-1, ade2-1, bar1::LEU2,* ***tlc1::TLC1-[10xMS2-IN]-natMX4-tADH1*** | This study |
| YV349, YV352 | *Mat A, ura3-1, leu2-3,112, trp1-1, ade2-1, bar1::LEU2,* ***his3-11,15::CreEBD-HIS3, tlc1::TLC1-[10xMS2-IN]-natMX4-tADH1*** | This study |
| YV359, YV360 | *Mat A, ura3-1, leu2-3,112, trp1-1, ade2-1, bar1::LEU2,* ***his3-11,15::CreEBD-HIS3, tlc1::TLC1-10xMS2*** | This study |
| YV355, YV356 | *Mat A, ura3-1, leu2-3,112, trp1-1, ade2-1, bar1::LEU2,* ***his3-11,15::CreEBD-HIS3, tlc1::TLC1-[10xMS2-IN]-natMX4-tADH1, kap122:: kanMX4*** | This study |
| *xpo1-1* | *Mat α, ade2-1, ura3-1, his3-11,15, trp1-1, leu2-3,112,* ***xpo1::LEU2, (pKW456 xpo1-1-HIS3)*** | (Stade *et al.*, 1997) |
| YV365, YV366 | *Mat α, ade2-1, his3-11,15, trp1-1, leu2-3,112,* ***xpo1::LEU2, (pKW456 xpo1-1-HIS3), ura3-1::CreEBD-URA3, tlc1::TLC1-[10xMS2-IN]-natMX4-tADH1*** | This study |
| *mex67-5* | *Mat A, ade2-1, ura3-1, his3-11,15, trp1-1, leu2-3,112,* ***mex67::HIS3, (pUN100 mex67-5-LEU2)*** | (Segref *et al.*, 1997) |
| YV385, YV386 | *Mat A, ade2-1, his3-11,15, trp1-1, leu2-3,112,* ***mex67::HIS3, (pUN100 mex67-5-LEU2),*** ***ura3-1::CreEBD-URA3, tlc1::TLC1-[10xMS2-IN]-natMX4-tADH1*** | This study |
| YV399,  YV400 | *Mat A, ade2-1, his3-11,15, trp1-1, leu2-3,112,* ***mex67::HIS3, (pUN100 mex67-5-LEU2),*** ***ura3-1::CreEBD-URA3, tlc1::TLC1-[10xMS2-IN]-natMX4-tADH1, rrp6::kanMX4*** | This study |
| Y11429 | *Mat A, his3Δ1, leu2Δ0, met15Δ0, ura3Δ0, mex67::mex67-5-kanMX6* | (Li *et al.*, 2011) |
| YV417,  YV418 | *Mat α, ade2-1, his3-11,15 trp1-1, leu2-3,112,* ***xpo1::LEU2, (pKW456 xpo1-1-HIS3), mex67::mex67-5- kanMX6, ura3-1::CreEBD-URA3, tlc1::TLC1-[10xMS2-IN]-natMX4-tADH1*** | This study |
| YV390, YV391 | *Mat A, ura3-1, leu2-3,112, trp1-1, ade2-1, bar1::LEU2,* ***his3-11,15::CreEBD-HIS3, tlc1::TLC1-[Sm2T-10xMS2-IN]-natMX4-tADH1*** | This study |
| YV403,  YV404 | *Mat α, ade2-1, his3-11,15, trp1-1, leu2-3,112,* ***xpo1::LEU2, (pKW456 xpo1-1-HIS3), ura3-1::CreEBD-URA3, tlc1::TLC1-[Sm2T-10xMS2-IN]-natMX4-tADH1*** | This study |

## References:

Li, Z. *et al.* (2011) ‘Systematic exploration of essential yeast gene function with temperature-sensitive mutants’, *Nature Biotechnology*. Nature Publishing Group, 29(4), pp. 361–367. doi: 10.1038/nbt.1832.

Lisby, M. *et al.* (2004) ‘Choreography of the DNA damage response: Spatiotemporal relationships among checkpoint and repair proteins’, *Cell*, 118(6), pp. 699–713. doi: 10.1016/j.cell.2004.08.015.

Segref, A. *et al.* (1997) ‘Mex67p, a novel factor for nuclear mRNA export. Binds to both poly(A)+ RNA and nuclear pores’, *EMBO Journal*, 16(11), pp. 3256–3271. doi: 10.1093/emboj/16.11.3256.

Stade, K. *et al.* (1997) ‘Exportin 1 (Crm1p) is an essential nuclear export factor’, *Cell*, 90(6), pp. 1041–1050. doi: 10.1016/S0092-8674(00)80370-0.
