# Supplementary material for: Telomerase biogenesis requires a novel Mex67 function and a cytoplasmic association with the Sm7 complex
Source: eLife. 2020 Oct 23;9:e60000. doi: 10.7554/eLife.60000 (PMC7644208; doi:10.7554/eLife.60000)
Supplement: Supplementary file 2. — The table includes the names, description and sources of all plasmids used in the study. The plasmids are referred to in the text using the names provided in the table. [file elife-60000-supp2.docx]

## Supplementary File 2. Plasmids used in the study

| **Plasmid** | **Description** | **Source** |
| --- | --- | --- |
| pEB36 | *URA3, TLC1-[MS2-IN]-natMX4 (pRS306)* | This study |
| pYV132 | *URA3, TLC1-[MS2-IN]-natMX4-tADH1 (pRS306)* | This study |
| pYV142 | *URA3, TLC1-[Sm2T-MS2-IN]-natMX4-tADH1 (pRS306)* | This study |
| pTW40 | *HIS3, pTDH3-CRE-EBD-tCYC1 (pRS303)* | (Verzijlbergen *et al.*, 2010) |
| pYV144 | *URA3, pTDH3-CRE-EBD-tCYC1 (pRS306)* | This study |

## References:

Verzijlbergen, K. F. *et al.* (2010) ‘Recombination-induced tag exchange to track old and new proteins’, *Proceedings of the National Academy of Sciences of the United States of America*, 107(1), pp. 64–68. doi: 10.1073/pnas.0911164107.
