# Supplementary material for: Telomerase biogenesis requires a novel Mex67 function and a cytoplasmic association with the Sm7 complex
Source: eLife. 2020 Oct 23;9:e60000. doi: 10.7554/eLife.60000 (PMC7644208; doi:10.7554/eLife.60000)
Supplement: Supplementary file 3. — The table includes the names, sequences and description of all oligonucleotides used in the study. The oligonucleotides are grouped in the table based on their application. The oligonucleotides are referred to in the text using the names provided in the table. [file elife-60000-supp3.docx]

## Supplementary File 3. Oligonucleotides used in the study

| **Name** | **Sequence (5’ to 3’)** | **Description** |
| --- | --- | --- |
| ***Strain construction/Cloning*** | | |
| qTLC1-F | caaatgtgccccgtacatcga | To amplify *TLC1-[MS2-IN]* cassette |
| TLC1 AvaI-R | cctatcagtaacactcggggaagg |  |
| YV395_KAP122_F1 | ttgaataccatagtaactagtctcttattcttagtttttgAGATTGTACTGAGAGTGCAC | To amplify *kap122::kanMX4* cassette |
| YV396_KAP122_R1 | aagaatagccttcaccctcagcggagaaattcttaacaaaCTGTGCGGTATTTCACACCG |  |
| *rrp6Δ*_for | catctgaagatagacgaaataggaacaacaaacagcttataagcacccaaAGATTGTACTGAGAGTGCAC | To amplify *rrp6::kanMX4* cassette |
| *rrp6Δ*_rev | gggagccataactccatgacacagatattcgattagatgaatttagaggtCTGTGCGGTATTTCACACCG |  |
| YV518_MEX67-237 | ccatgcccacttgcctttcgtagagg | To amplify *mex67-5-kanMX6* cassette |
| YV519_MEX67+278 | ttggtactctgattccggagcctggc |  |
| ***Southern/northern blotting*** | | |
| TLC1-AflII-F | cctttcttaagcatcggttagg | To amplify TLC1-specific probe for Southern blot (with TLC1 AvaI-R) |
| CEN4 probe-F | atgctgtctcaccatagagaat | To amplify CEN4-1.5 kb probe for recombination Southern blot |
| CEN4 probe-R | cgctcctaggtagtgcttt |  |
| TLC1 RT-F2 | agaacgtcagggaacatgagt | To amplify TLC1-specific probe for northern blot |
| TLC1 RT-R2 | tgtacggggcacatttgga |  |
| non-aminoallyl MS2 | tgggtgatcctcatgttttctagagtcgacgtgcagacatgggtgatcctcat | Oligonucleotide MS2-specific probe for northern blot |
| U1 | agcgcatgtttgatcagtagg | Oligonucleotide U1-specific probe for northern blot |
| Ch IV-R | ctcattcgaatccatacgacc | To amplify CEN4-1.6 kb probe for teloblot |
| Ch IV-L | gtacctcggtttagttaagcg |  |
| ***FISH*** | | |
| TLC1-1^1^ | t*gcgcacacacaagcat*ctacactgacaccagcat*actcgaaattctt*tg | Oligonucleotide TLC1-specific FISH probes |
| TLC1-2^1^ | ct*aataaacaatt*agctgtaacatt*tgtgtgtggggt*gtggtgatggt*aggc |  |
| TLC1-3^1^ | tt*ccagagttaacgat*aagatagacat*aaagtgacagcgct*tagcaccgt*c |  |
| TLC1-4^1^ | ttacgt*tcttgatctt*gtgtcattgtt*cagttactgat*cgcccgcaaacct* |  |
| TLC1-5^1^ | tgcat*cgaaggcat*taggagaagt*agctgtgaat*acaacaccaagat*tca |  |
| MS2^2^ | t*gggtgatcctcatgtt*ttctagagtcgacgt*gcagacatgggtgatcct*cat | Oligonucleotide MS2-specific FISH probe |
| ***RT-ddPCR, qPCR*** | | |
| TLC1.AB.qsc.F3 | ccaagcggaaggaaccgtgt | To detect untagged TLC1 |
| TLC1.A.lna.qsc.R4 | c^c^aa^a^aa^ttatctaaatgcatataac |  |
| TLC1.B.qsc.R1 | gtgatcctcatgtttctggccatataac | To detect tagged TLC1-MS2 (with TLC1.AB.qsc.F3) |
| TLC1.AB.qsc.F1 | cggttaggtttgcgggcgat | To detect total TLC1 |
| TLC1.AB.qsc.R1 | acacggttccttccgcttgg |  |
| TLC1_G_for1 | ttaggtttgcgggcgatcagt | For ChIP at the *TLC1* locus |
| TLC1_G_rev1 | acggttccttccgcttggaaa |  |
| U1 RT F2 | ttggtcacacacacatacgg | For ChIP at the *U1* locus |
| U1 RT R2 | ggtgtcaaacttctccaggc |  |

^1^ (Gallardo *et al.*, 2008)

^2^ (Bajon, Laterreur and Wellinger, 2015)

* - aminoallyl modified-T conjugated to the Cyanine dye

^N - locked nucleic acid (LNA)

## References

Bajon, E., Laterreur, N. and Wellinger, R. J. (2015) ‘A Single Templating RNA in Yeast Telomerase’, *Cell Reports*. The Authors, 12(3), pp. 441–448. doi: 10.1016/j.celrep.2015.06.045.

Gallardo, F. *et al.* (2008) ‘TLC1 RNA nucleo-cytoplasmic trafficking links telomerase biogenesis to its recruitment to telomeres’, *EMBO Journal*, 27(5), pp. 748–757. doi: 10.1038/emboj.2008.21.
